# Case Report: 3D-Printed Prosthesis for Limb Salvage and Joint Preservation After Tibial Sarcoma Resection

**DOI:** 10.3389/fsurg.2022.873272

**Published:** 2022-05-27

**Authors:** Zehao Guo, Ran Zhang, Yukang Que, Bo Hu, Shenglin Xu, Yong Hu

**Affiliations:** Department of Orthopedics, The First Affiliated Hospital of Anhui Medical University, Hefei, China

**Keywords:** 3D-printed prosthesis, tibia, chondrosarcoma, joint-preserving surgery, limb salvage

## Abstract

**Introduction:**

Reconstruction of massive tibial defects in ankle joint-preserving surgery remains challenging though biological and prosthetic methods have been attempted. We surgically treated a patient with only 18-mm distal tibia remaining and reconstructed with a unique three-dimensional printed prosthesis.

**Case Presentation, Intervention, and Outcomes:**

A 36-year-old male presented to our clinic with complaints of gradually swelling left calf and palpable painless mass for five months. Imageological exam indicated a lesion spanning the entire length of the tibia and surrounding the vascular plexus. Diagnosis of chondrosarcoma was confirmed by biopsy. Amputation was initially recommended but rejected, thus a novel one-step limb-salvage procedure was performed. After en-bloc tumor resection and blood supply rebuilding, a customized, three-dimensional printed prosthesis with porous interface was fixed that connected the tumor knee prosthesis and distal ultra-small bone segment. During a 16-month follow-up, no soft tissue or prosthesis-related complications occurred. The patient was alive with no sign of recurrence or metastasis. Walking ability and full tibiotalar range of motion were preserved.

**Conclusions:**

Custom-made, three-dimensional printed prosthesis manifested excellent mechanical stability during the follow-up in this joint-preserving surgery. Further investigation of the durability and rate of long-term complications is needed to introduce to routine clinical practice.

## Introduction

The tibia is the second most frequently affected bone in terms of primary bone sarcomas and it may also be involved in metastatic diseases (1). While malignant tumors affecting the distal tibia are relatively rare (2). There is currently no consensus on the reconstruction of this site. Compared with arthrodesis and arthroplasty, joint-preserving surgery, being technically more demanding, has yielded a better functional outcome without jeopardizing the oncological principles (3). Following extensive tumor resection, though implantation of metallic prosthesis renders early ambulation and decreases the rate of fracture, which is superior to the allograft (4, 5), rigid fixation of the short bone residue is challenging with short-stemmed endoprostheses and the high-stress concentration at the contact interface heralds the loosening probability. Extracortical plates, having the ability to be integrated to form a new fixation system, have been most widely used and showed promising preliminary results (6, 7). The retained natural joints and ligaments enable normal movement. And limb function is maintained with the achievement of solid osseointegration at the junction site.

Cases of total tibial reconstruction are rare and only four have been reported where ankle joints were sacrificed with either hindfoot fusion nail or metal tibiotalar components. Compared with a large segment allograft, endoprosthesis was thought to be a more appealing choice (8–11). The introduction of image processing and three-dimensional (3D) printing technology has ushered in a new era in oncological orthopedics for patient-specific reconstruction. Although there are concerns regarding the lack of international standards and evaluation systems, ability to clean lattice structures, and mechanical strength (12), 3D-printed trabecular meshwork, featured by excellent biocompatibility and intrinsic osteogenic property (13, 14), has been applied in prosthesis fabrication.

We present here a customized, 3D-printed prosthesis with porous contact surface and extracortical plates for the reconstruction of a massive defect of almost the whole length of the tibia with the preservation of the ankle joint. There is no similar limb salvage and joint-sparing approach reported before.

## Case Presentation

**Figure 1:**
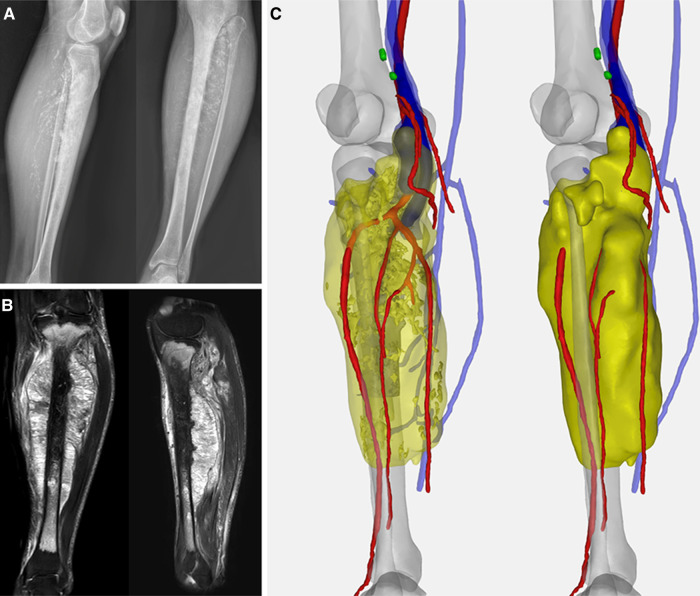
(**A**) Preoperative X-ray indicated the spindle-shaped lesion; (**B**) preoperative MRI showed tumor involving the whole length of the tibia with a heterogeneous hyperintense signal on T1-weighted and hypointense signal on T2-weighted images. (**C**) Computer-aided, 3D angiography illustrated the anterior tibial artery, posterior tibial artery, peroneal artery, and vena comitans of the affected side were encompassed.

A 36-year-old male was referred to our clinic with complaints of gradually swelling left calf and palpable painless mass since five months ago. Plain radiography revealed irregular punctate calcification within the soft mass silhouette. Magnetic resonance imagery (MRI) images indicated that the lesion formed a large spindle soft tissue mass with a size of 194.3 × 81.8 × 60.8 mm. Sagittal images demonstrated the breach of the tibial plateau and the range of abnormalities was about 300 mm with 28 mm distal tibia intact (Figure 1). The pathological diagnosis of chondrosarcoma was confirmed by computed tomography (CT)-guided core needle biopsy at the local hospital. Staging investigations including chest CT and positron emission tomography-computed tomography (PET-CT) found no evidence of metastasis.

According to the computer-aided, 3D angiography of the lower extremity, the anterior tibial artery, posterior tibial artery, peroneal artery, and vena comitans were wrapped by tumors. Thus, mid-thigh amputation was initially recommended. But the patient firmly refused for psychological and social reasons.

Due to the resistance of chondrosarcoma to chemotherapy, a one-step surgery was planned that included joint-preserving tumor resection, contralateral great saphenous vein grafts aided reperfusion of the distal extremity, and prosthetic reconstruction of the bone defect. Considering the massive osseous defect that would be present, the application of customized, 3D-printed implant and patient-specific guide (PSG) was opted.

## Prosthesis Design and Fabrication

**Figure 2:**
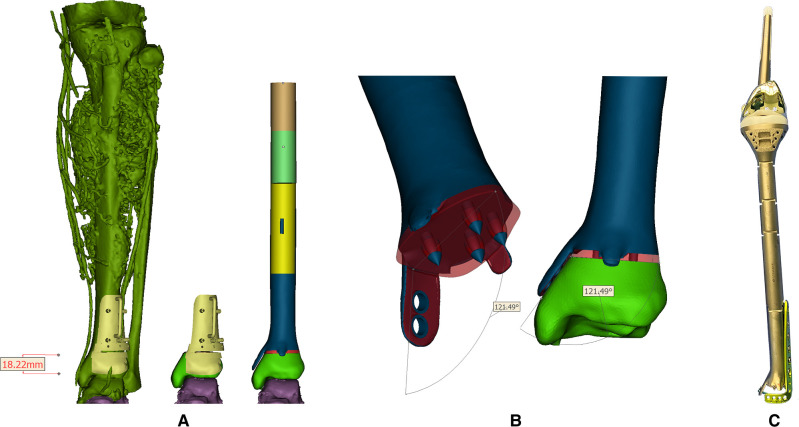
(**A**) Reconstructed tumoral 3D model and the reconstruction plan by a custom-made prosthesis after tumor resection with the aid of PSG. Blue, red parts, and the PSG were 3D-printed while the leftover shaft was fabricated by traditional manufacturers; (**B**) Design of the 3D-printed prosthesis equipped with extracortical plates and porous structure. Red region: porous structure; blue region: solid structure; (**C**) The real picture of the reconstruction system.

CT scan data in DICOM format was imported into Mimics software (16.0, Materialise Inc., Leuven, Belgium) to reversely construct the 3D model. A uniplanar bone resection was employed. Osteotomy plane was defined based on MRI images and measured by the distance from the adjacent joint line with an extra 10-mm tumor-free margin. PSG was designed to be fully fit with the ventral tibia and then fabricated from nylon powder using selective laser sintering technique. After anatomic placement, the PSG could be secured by K-wires via drill sleeves. A cutting platform that provided the osteotomy trajectory and confined the oscillating saw was incorporated (Figure 2A). Size of the tibia and osseous defect was measured. For the leftover length of customized components, the height of the standard modular plateau was subtracted. The distal third of the prosthesis was manufactured by 3D printing while the rest was fabricated by traditional subtractive manufacturing (Chun Li Zheng Da, Co, Ltd, Beijing, China) with Ti6Al4V and connected by snap-fit attachment. Trabecular pores were fabricated by electron beam melting (EBM) technique (mean porosity, 70%; mean pore size, 600 µm; wire diameter, 600 µm) at bone-contacting surface to promote osteointegration according to a computer-aided design (CAD) model (Figure 2B). The metallic alloy powders were melted by the energy emission from the electron beam of a tungsten filament and printed layer by layer. The surface of the product was then subjected to postprocessing.

The design stemmed from the concept of an essentially extensive proximal tibial replacement incorporating an oncologic knee prosthesis. Mini-extracortical plates were located dorsally and ventrally at the distal margin to prevent sagittal angulation during the early stage of bone growth. One larger extracortical plate extending medially corresponded neatly to the dimensions and shape of the medial malleolus that acted as the main anchorage tool of the implant to the remaining bone segment. Due to the limited residual bone stock and sectional area of the distal tibia, four spikes were used to increase bone contact and prevent excessive medullary violation. The tip of each spike was solid for press-fitting onto the cancellous bone in a cementless manner, while the base was trabecular, fusing with the surrounding porous structures. Screw holes were customized on the middle and lower third of the prosthesis to be fitted with an off-the-shelf L-shape tibial plate (Figure 2C).

## Surgical Technique

**Figure 3:**
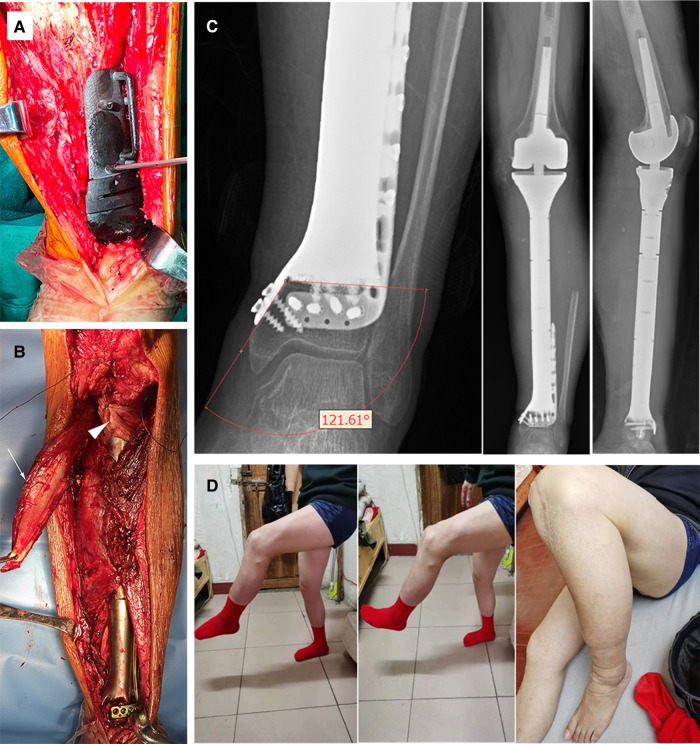
(**A**) Intraoperative utilization of PSG to ensure precise osteotomy; (**B**) The anatomic implantation of the prosthesis. White arrowhead: medial gastrocnemius flap; triangular arrowhead: double-surface reconstruction of the knee extension mechanism with an allogenic tendon. (**C**) X-ray of the lower limb 12 months after surgery. The angle of the medial plate was about 121 degrees, which was consistent with the design. No sign of ischemic necrosis or collapse of the distal ultra-small bone fragment was observed. (**D**) The active knee range of motion (ROM) was 0–105 degrees and the patient sustained active ankle ROM of dorsal flexion 0–15 degrees, and plantar flexion 0–45 degrees at the latest follow-up.

An extensive anteromedial approach was applied. Resection margin of soft tissue was determined according to the MRI images. The PSG, fitting anatomically on the distal tibia, was firmly stabilized with K-wires to facilitate planned osteotomy (Figure 3A). Then the tibia was elevated to expose posterior structures for en-bloc tumor resection. The tibial nerve and common peroneal nerve, being located outside the border of the tumor, were left intact. Three nutrient arteries and vena comitans were transected while the superficial veins were preserved. The tumor thrombus was removed en bloc with the popliteal vein. The length of the posterior tibial artery defect was measured and the great saphenous vein contralaterally located was harvested as a graft to rebuild the blood perfusion. The knee joint was reconstructed with a rotating-hinge modular system, followed by connection to the custom-made prosthesis. Afterwards, the plate and screws were inserted to strengthen the fixation at the ultra-small tibial stump. Extensor apparatus was restored by a double-surface mechanism. Medial gastrocnemius flap was transposed to wrap the proximal prosthesis. Soleus muscle was horizontally stretched and sutured with anterior tibial muscle fascia to cover the middle portion (Figure 3B). Pulsatile lavage with saline solution was used to rinse the surgical area and the wound was closed in layers. Histological analysis showed an R0 resection.

## Postoperative Management

The affected limb was fixed with an extension splint for 4 weeks after the operation. Antithrombotic prophylaxis was performed using low molecular heparin calcium with a dose of 4,000 IU per day. A third-generation cephalosporin was given for 14 days, followed by an oral combination of levofloxacin and rifampicin for one month. Progressive axial weight-bearing exercise was carried out 2 weeks after the operation with a brace. Postoperative X-rays were compared with blueprints to confirm the precision of manufacture and angle stability of the prosthesis (Figure 3C).

## Outcomes

Chest CT was taken every 3 months and contrast-enhanced CT of the lower limbs was taken one year after. The patient underwent evaluations including physical examinations and plain radiography before discharge and 1, 3, 6, 12, and 16 months thereafter.

The patient reported no palpable mass or discomfort except for mild edema following long-term standing and was alive without evidence of local recurrence or distant metastasis. No disruption of prosthetic components or aseptic loosening occurred. And no evidence of ischemic necrosis or collapse of the ultra-small bone fragment was observed.

Functional evaluation determined at the 16-month mark by Musculoskeletal Tumor Society (MSTS) score (15) was 5, 4, 5, 5, 4, 5 points each for pain, function, emotional acceptance, supports, walking ability, and gait, with a total score of 28 (93%). The Toronto Extremity Salvage Score (TESS) (16) was 90. The patient resumed his full-time occupation as a middle school teacher, and his previously active lifestyle was maintained, being able to ambulate without discernible limp, stand from a seated position, and climb stairs unassisted (Figure 3D and Supplementary Video S1).

## Discussion

There were some limitations in this report. First, the relatively short follow-up period confined our results. Observation of the durability and long-term complications is needed. Although the functional and oncological outcome was satisfactory, we recognize that this case provided an alternative to amputation and mid-thigh amputation still remains the most reliable strategy for achieving intermediate results. Second, the nature of titanium alloy made it difficult to monitor the degree of osseointegration for making CT and MRI images around the implant distorted unless an amputation specimen is retrieved (17, 18). Even though T-SMART scanning was reported to be one of the few methods (19), it could not provide direct evidence of bone ingrowth due to the size of the micropore.

Treatment of juxta-articular bone tumor remains a challenge. Joint-preserving surgery may provide a similar risk of complications when compared with reconstructions sacrificing joint (20), with the added theoretical advantages of better proprioception, maintained joint function, and fewer complications related to polyethylene liner (21) in carefully selected patients. Likewise, early ambulation is encouraged, reducing complications caused by long-term bed rest. But a tumor-free boundary should be guaranteed for not jeopardizing oncologic outcomes. Intralesional surgery margins have been shown to be responsible for local recurrence and poor outcomes (22). With higher accuracy of imaging technology in delineating tumors, closer margins are obtained, allowing more joint- or physical-sparing tumor resections. The criteria for joint-preserving surgery ranged from 1 to 5 cm of residual bone since 1999 (23–27).

For lesions invading the metaepiphysis and the distance between the tumor edge and articular cartilage is less than 1 cm, joint-salvage surgery seemed to be contradicted. A novel adjuvant technique consisting of argon-based cryoablation and transepiphyseal osteotomy was proposed under the circumstances (28). The short-term outcome was excellent while osteonecrosis of varying degrees was observed in all residual epiphyses, which was considered a precursor to microcollapse, joint instability, and further degeneration. Sufficient subchondral bone stock is conducive to reducing the occurrence of complications (29). The concept “ultra-critical sized bone defect” was proposed to depict tumor-induced bone defects which pose challenges for using off-the-shelf prostheses that can reliably grasp small joint- or physis-containing fragments (14). 3D-printed prostheses containing short stems with cross screws fixation were designed to fulfill the requirement. And no mechanical complications occurred during a mean follow-up of 27.6 months. Following extensive segmental bone resection, endoprostheses inherently have an increased risk of loosening due to limited bone/metal or bone/cement interface. It has been suggested that extracortical plates, being supposed to be incorporated by the newly expanded cortex to form a new fixation system, offered an alternative. The addition of extracortical plates to short stems was not inferior to standard length medullary stems and minimized aseptic failure (30). When the available canal length precluded the use of stems, prostheses without intramedullary stems also manifested promising preliminary results with the aid of extracortical plates (7, 27).

In the cases of lesions in the distal tibia, the outcome of ankle endoprosthesis was not ideal due to talar collapse, infection, aseptic loosening, and a diminished maximum angle of plantar flexion (5, 23, 31). Feng et al. (32) reported a 3D-printed distal tibial prosthesis of 9.5 cm in length. Several screws were used to fix the metal ankle mortise to the native talus. Life expectancy of the jointless prosthesis was supposed to be better, whereas the tibiotalar joint was sacrificed, leaving only micromotion of dorsal and plantar flexion. Although arthrodesis is a more reliable method to acquire a stable and durable construct compensating for transection of peri-ankle ligaments, limb function may be severely affected given the loss of sagittal and hindfoot motion (33).

It is challenging to perform joint-preserving surgery using a prosthesis for massive bone defects of total tibial length combined with ultra-short distal residue. The macroporous structure, whose size is thought to be optimal at 640 mm, facilitates interlocking between host bone and implants by providing room for newborn tissue, which is conductive to biological fixation (34). However, traditional manufacturing methods, such as powder sintering, plasma spray coating, and foam fabrication, have the inherent inability to precisely control the pore parameters (35). With the in-depth development of additive manufacturing technology, also known as 3D printing technology, the customized implant can be manufactured with metal alloys, and the capacity to be resistant to fatigue, corrosion and fracture can achieve the clinical requirements (36). The lattice structure can be altered by changing the CAD model to modify mechanical properties and biological performance. The patient-individualized and anatomy-imitating implants in complex geometric and biomechanical sites have shown promising results in osteoarticular and intercalary reconstructions following limb- and joint-salvage surgeries (13, 37). Considering several advantages of 3D-printed implants, we designed a custom-made, uncemented prosthesis. By comparing the blueprint and postoperative images, 3D printing technology showed excellent angular and dimensional accuracy in manufacturing, providing perfect bony reconstruction. To the best of our knowledge, reports of tibiotalar joint-preserving surgery using a metal prosthesis are extremely rare. And there is no reported application of custom-made, 3D-printed implant to restore the continuity of the tumor knee prosthesis and distal short segment as well as the outcome so far. Follow-up was conducted for a period of 16 months. A satisfactory outcome was obtained and no complications occurred.

3D-printed implants with the highly cancellous surface have developed biological fixation in the orthopedic area. A titanium interface with an interconnected porous structure was produced using the EBM technique, giving the requisite strength while enabling bone growth deep within the pores (17, 19, 38). However, primary mechanical stability is deemed an essential precondition for successful osseointegration (14). To meet the requirement for superior pullout strength and stability, screw holes on the middle and lower third of the prosthesis were customized to be perfectly fitted with a dismountable L-shape tibial plate. Once provisional rotational stability is established, cementless fixation may be advantageous due to the preservation of the bone inducibility and bioactivity, which results in a low rate of aseptic loosening (39). Preoperative imaging modalities can accurately reflect the extent of the tumor. Nonetheless, the tumor margin is difficult to be transposed during operation. 3D-printed PSG served as an alternative in the precise planning and execution of joint-preserving tumor resection. With the PSG perfectly conforming to anatomical structures, it was unnecessary to measure the distance during the operation, therefore, reducing the risk of manual errors and the operation time, especially in complex multiplanar bone resections. And the introduction of computer navigation techniques to verify the correct placement of PSG intraoperatively rendered a more precise safe margin (40). However, there are concerns about 3D printed prostheses and PSGs before widespread clinical use. The close-loop standard evaluation system for implants is lacking though patients were informed of the unknown risks. The carcinogenicity and genotoxicity associated with degradation needs keeping track of the long-term outcomes. And it is imperative to establish appropriate sterilization methods and standards, especially for porous structure. Improper sterilization adversely affects prostheses and can result in uncontrollable infection and a failed reconstruction (41). Moreover, the high cost of equipment acquisition, maintenance, and a high threshold for the use of software seriously limit its popularity.

Tissue coverage is the key to extending the life of prostheses and improving the prognosis. The medial gastrocnemius flap, being advantageous in promoting collagen deposition and tissue ingrowth along with the characteristics of versatility and malleability to fill dead space, was proposed in endoprosthetic tibia replacement to decrease infection rates (42, 43). Restoring the continuity of patellar tendon attachment to the tibial component was of paramount importance to achieve functional reconstruction, rather than being confined to only anatomical and cosmetic. By using a double-interface reconstructive method, the material/tendon interface is effectively repaired in a tension-free manner with tight sutures and immobilization (44, 45). In this case, with the allogenic tendon encompassing the proximal prosthesis, tension applied during extension movement was converted to friction, thereby preventing local wear caused by stress concentration. And the friction increased as scar tissue formed to sustain the bond at the contact. Following en bloc resection with clear surgery margins, the muscles and tendons were preserved to the greatest extent. Therefore, ankle strength and toe movement were maintained along with a favorable oncological outlook. A consideration was also given to devascularization of the bone segment. The ventral tibia was sufficiently exposed to install the PSG and plate while the blood to the dorsal and lateral surface was completely preserved. The bone-contacting surface of the extracortical plates was porous, facilitating bone ingrowth and integration while protecting the periosteal blood supply. In addition, the striking structure on the distal flat surface decreased the violation of the epiphyseal cancellous bone, thereby preventing the excessive destruction of the blood perfusion. We hope all the factors mentioned above would help diminish the risk of bone necrosis.

## Conclusion

This case report first presents a novel limb- and joint-preserving alternative to amputation for patients with chondrosarcoma invading the entire length of the tibia and vascular bundle. The computer-assisted design, 3D-printed tibial prosthesis equipped with extracortical plates and porous contact surface, along with a dismountable plate, exhibited the excellent capacity to grasp the ultra-short residual bone of distal tibia in the short-term period. Longer follow-up is needed to verify the durability of the prosthetic and soft tissue reconstruction.

## Data Availability

The original contributions presented in the study are included in the article/Supplementary Material, further inquiries can be directed to the corresponding author/s.
