# Supplementary material for: Case Report: 3D-Printed Prosthesis for Limb Salvage and Joint Preservation After Tibial Sarcoma Resection
Source: Front Surg. 2022 May 27;9:873272. doi: 10.3389/fsurg.2022.873272 (PMC9195185; doi:10.3389/fsurg.2022.873272)
Supplement: Supplementary file 1 [file Data_Sheet_1_v1.pdf]

CARE case report guidelines

CARE Checklist of information to include when writing a case report

(cc) BY-NC-ND

Topic	Item	Checklist item description	Reported on Line
Title	1	The diagnosis or intervention of primary focus followed by the words "case report"	1
Key Words	2	2 to 5 key words that identify diagnoses or interventions in this case report, including "case report"	10
Abstract (no references)	3a	Introduction: What is unique about this case and what does it add to the scientific literature?	14-17
	3b	Main symptoms and/or important clinical findings	18-27
	3c	The main diagnoses, therapeutic interventions, and outcomes	18-27
	3d	Conclusion—What is the main "take-away" lesson(s) from this case?	28-30
Introduction	4	One or two paragraphs summarizing why this case is unique (**may include references**)	44-51
Patient Information	5a	De-identified patient specific information.	57-69
	5b	Primary concerns and symptoms of the patient.	57-58
	5c	Medical, family, and psycho-social history including relevant genetic information	NA
	5d	Relevant past interventions with outcomes	62-65
Clinical Findings	6	Describe significant physical examination (PE) and important clinical findings.	60-69
Timeline	7	Historical and current information from this episode of care organized as a timeline	57-74
Diagnostic Assessment	8a	Diagnostic testing (such as PE, laboratory testing, imaging, surveys).	57-69
	8b	Diagnostic challenges (such as access to testing, financial, or cultural)	NA
	8c	Diagnosis (including other diagnoses considered)	62-63
	8d	Prognosis (such as staging in oncology) where applicable	62-65
Therapeutic Intervention	9a	Types of therapeutic intervention (such as pharmacologic, surgical, preventive, self-care)	70-124
	9b	Administration of therapeutic intervention (such as dosage, strength, duration)	70-124
	9c	Changes in therapeutic intervention (with rationale)	NA
Follow-up and Outcomes	10a	Clinician and patient-assessed outcomes (if available)	126-138
	10b	Important follow-up diagnostic and other test results	126-138
	10c	Intervention adherence and tolerability (How was this assessed?)	126-138
	10d	Adverse and unanticipated events	129-130
Discussion	11a	A scientific discussion of the strengths AND limitations associated with this case report	140-246
	11b	Discussion of the relevant medical literature **with references**.	140-246
	11c	The scientific rationale for any conclusions (including assessment of possible causes)	140-246
	11d	The primary "take-away" lessons of this case report (without references) in a one paragraph conclusion	248-253
Patient Perspective	12	The patient should share their perspective in one to two paragraphs on the treatment(s) they received	129-130, 130-138
Informed Consent	13	Did the patient give informed consent? Please provide if requested	Yes ☑ No ☐
